# Parents’ experiences of telephone nursing when their child has a fever: a mixed method study

**DOI:** 10.1186/s12912-026-04585-0

**Published:** 2026-03-30

**Authors:** Emma Westin, Anders Svensson, Ingrid L. Gustafsson, Carina Elmqvist, Märta Sund Levander

**Affiliations:** 1 Department of Health and Caring Sciences, Faculty of Health and Life Sciences, Linnaeus University, Växjö, Sweden; 2 Department of Paediatrics, Region Kronoberg, Växjö, Sweden; 3 Centre of Interprofessional Collaboration within Emergency Care (CICE), Växjö, Sweden; 4 Department of Caring Sciences, Faculty of Caring Science, Worklife and Social Welfare, University of Borås, Borås, Sweden; 5 Department of Research and Development, Region Kronoberg, Växjö, Sweden; 6 Department of Health, Medicine and Caring Sciences, Medical Faculty, Linköping University, Linköping, 58185 Sweden

**Keywords:** Communication, Fever, Parent experiences, Mixed-method, Nurse-parent interaction, Telenursing

## Abstract

**Background:**

When fever causes concerns for parents, they can contact Swedish Healthcare Direct (SHD), i.e. the telephone nursing (TN) service. However, ensuring satisfaction, consensus and trust is crucial for parental reassurance and adherence to the advice. There is limited research investigating parents’ views of TN, especially as regards children with fevers. The study aimed to explore and describe parents’ experiences of telephone nursing when their child has a fever.

**Method:**

The study uses a convergent mixed-method design. Parents who contacted the TN service regarding their child’s fever were invited to participate. Quantitative data were collected in 113 questionnaires and analysed with descriptive statistic (n, %, median and interquartile range). Qualitative data were collected in 12 semi-structured interviews and analysed with manifest content analysis. Quantitative and qualitative data were analysed separately and then integrated and presented in a way that compares similarities and differences. Finally, an overall interpretation took place.

**Results:**

Descriptive statistics showed that the majority of parents had both high expectations of the telenurses and also high parental satisfaction with the TN service. The qualitive analysis identified three themes; seeking answers, getting a caring conversation, and getting an assessment and guidance. The integrated results show that the qualitative findings both confirmed and extended the questionnaire responses. The interaction with the telenurse plays an important role and sometimes seems to play an even larger role in parents’ satisfaction with a call than the actual advice they received. The parents describe being cared for by someone with an empathetic response including a reassuring tone of voice. Although the waiting times can be perceived as too long, the lengths of the calls are generally described as appropriate, not because of the duration itself, but because the telenurses seem calm and do not end conversations prematurely.

**Conclusion:**

The findings show that telephone nursing encompasses both advice and the telenurse-parent interaction surrounding its provision. Each of these aspects are important in creating satisfied and reassured parents. The advice must be clear, consistent and preferably include safety netting. At the same time, the telenurse must listen, validate and take the parent’s concerns seriously.

**Clinical trial number:**

Not applicable.

## Background

Fever is a symptom that often causes concerns among parents. It is a common reason why they seek healthcare for their children [1–3], as it can be difficult to assess a child’s symptoms and severity of illness, and parents thus need both informational and emotional support [4]. One accessible path to healthcare is telephone nursing (TN). This service is now available in numerous countries, among them Sweden, Denmark, the Netherlands, United Kingdom and Canada, though the manner in which it is organized varies [5].

Swedish Healthcare Direct (SHD) was launched in 2003 as a TN service that offers the population easily accessible and safe healthcare advice provided by registered nurses (hereinafter referred to as “telenurses”). Since 2013, the service has been available around the clock regardless of where the caller lives. It is the largest healthcare service in Sweden and is considered to be a form of first-line care. Overall, SHD receives approximately 5.5 million calls per year [6]. The advice provided by the service’s telenurses comprises symptom assessment, healthcare guidance, and referrals to other parts of the healthcare system. This process is guided by a symptom-based computerised decision support tool based on national guidelines and Swedish medical practice. This ensures more reliable and consistent assessment [7]. In TN, the communication between the caller and the telenurse is key, as interactions often consist solely of verbal exchange [6].

TN has been shown to enable the caller to remain at home and follow self-care advice, even if their initial intention was to seek further care at a healthcare facility [8]. As regards parents calling on behalf of their children, approximately 30–50% are advised to continue to monitor their child at home [2, 8]. The research of Sundberg et al. [9] indicates that when parents of children under ten years of age received telenursing advice to stay at home, 90% followed that advice and did not visit a healthcare facility within 72 hours [9]. This shows that TN plays a significant role in paediatric healthcare utilization [8, 9].

There is also a connection between interaction and how the advice is received [10–12]. Gustafsson et al. [8] found a correlation between received self-care advice and lesser satisfaction in adult callers, including parents. This suggests the existence of remaining needs that have not been met. One main factor in caller satisfaction is reassurance. In TN, this can be provided by creating a trusting relationship [8]. Kaminsky et al. [10] underscore that even though some parents state that they follow the telenurses’ advice regardless of what they are told, others will only follow the advice if it concurs with their own sense of what is correct [10]. If parents feel that a telenurse cares, listens and understands, this can increase their confidence in the advice they are given and decrease their desire for a second opinion [10, 11]. TN thus has the potential to create satisfied and reassured parents, but there is a complexity in the individual parent’s needs during the encounter. It is therefore important to explore how the parent-telenurse interaction during TN can be reinforced from the parent’s perspective. However, there is a dearth of research that investigates parents’ views of TN [6, 10], especially as regards fever. Therefore, this study aims to explore and describe parents’ experiences of telephone nursing when their child has a fever.

## Method

### Design

**Fig. 1:**
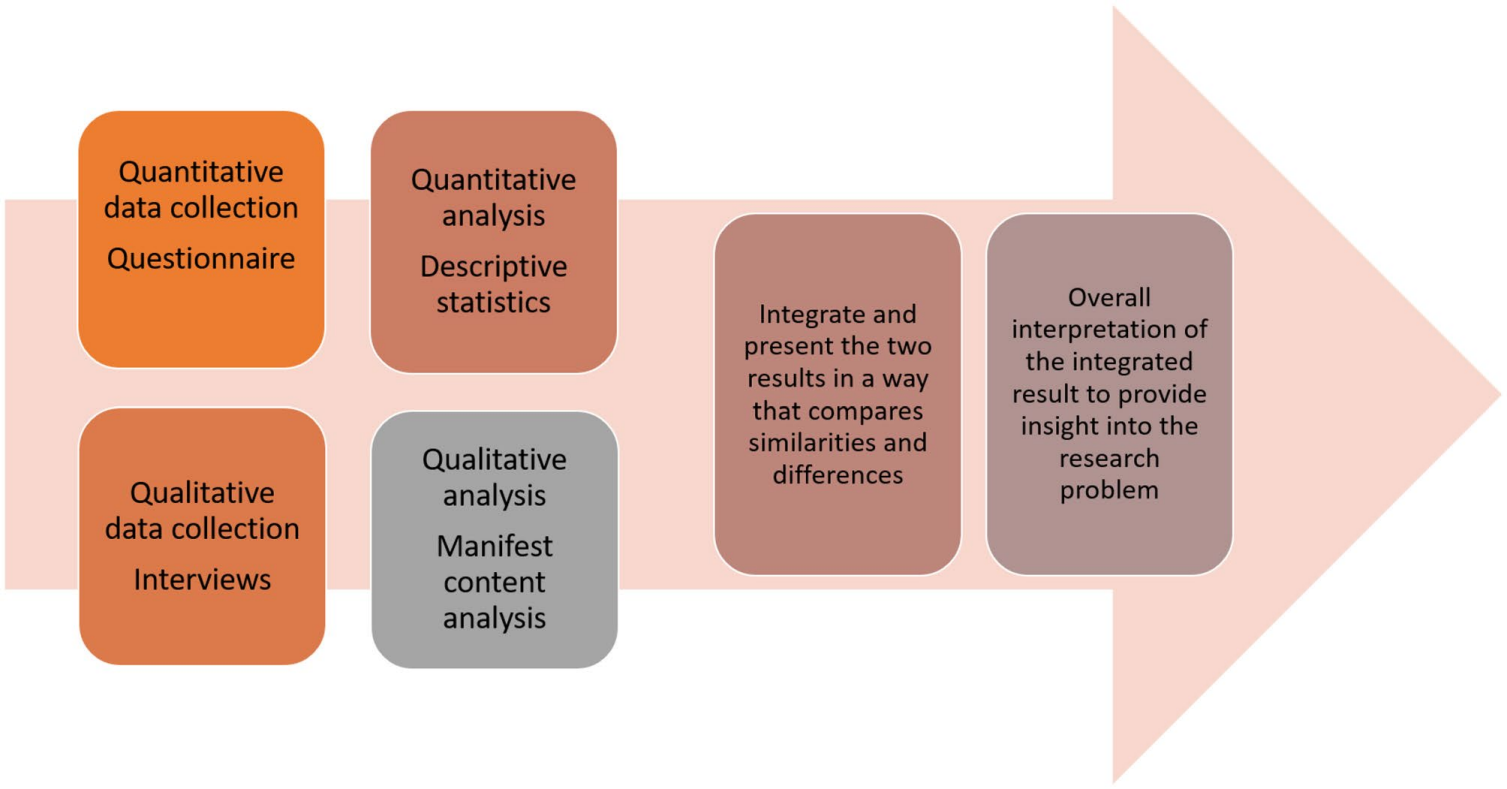
Convergent mixed-method design in accordance with the approach of Creswell & Plano Clark [13]

A convergent mixed-method design following the approach of Creswell and Plano Clark [13] was used. The mixed-method aspect of the design consists of four phases. In the first phase, quantitative and qualitative data are collected individually; this means that both data collections are planned and made simultaneously, but are not influenced by each other’s results. In the next phase, quantitative and qualitative data are analysed separately. In the third phase, the two results are integrated and presented in a way that compares similarities and differences. Finally, an overall interpretation takes place in Phase 4; see Fig. 1. This design is used when addressing research questions that benefit from multiple methodological approaches, especially in the study of complex phenomena encompassing both quantifiable dimensions and subjective experiences [13].

### Setting and sample

The study was conducted at SHD in three regions in southern and central Sweden, over a two-year period (spring 2022 to spring 2024), except for one region that withdrew after one year. The first author contacted the department head at each workplace, who then sent out information about the study to their telenurses. The first author also attended workplace meetings to provide information and answer questions. A consecutive sampling method was used, whereby all participants who met the inclusion criteria were recruited consecutively over the two-year study period. Each year were divided into three periods: spring, autumn and winter. Inclusion criteria were Swedish-speaking parents of children under six years of age who sought TN advice regarding their child’s fever.

### Data collection

Parents were initially invited by the telenurses to complete a questionnaire after the call. In this questionnaire, the parents could also consent to be contacted for information about an individual follow-up interview. There was no compensation offered for participating in the interview.

#### Quantitative data collection

The Telenursing Interaction and Satisfaction Questionnaire (TISQ) [14] was sent out to parents who had agreed to take part in the study by e-mail or SMS (according to the parent’s preference), together with a written information letter regarding voluntary participation, consent and confidentiality, most often within a week after the call to SHD. The information letter stated that the parents gave informed consent by answering the questionnaire.

The TISQ were developed by Mattisson et al. [14] in order to measure the callers’ (patient/relative) satisfaction with their interaction with the telenurse during their TN interaction and is according to Mattisson et al. [14] based on the Interaction Model of Client Health Behaviour (IMCHB) by Cox. The TISQ has been found to yield good content validity within the context of SHD and moderate to good test-retest reliability (ICC = 0.39-0.84), internal consistency was not tested. The TISQ contains of a total of 60 questions divided into four separate sections (A, B, C, D; see Table 1) [14], of which 25 (Section B) pertain to the Telenursing Interaction and Satisfaction Scale (TISS). The remainder of the questions describe demographic facts about the caller and their previous healthcare experiences, as well as their emotional state and expectations prior to the interaction. Some questions describe attributes of the call, such as waiting time and when the call took place. For further information about the TISQ questions, see Mattisson et al. [14].

**Table 1:** Overview of the questionnaire’s sections, according to Mattisson et al. [14]

Section	Explanation
A.	Questions about the caller’s assessment of the situation and expectations prior to the call.
B.	Questions about perceptions of the interaction with the telenurse according to the four subscales of the TISS – health information, professional-technical competence, affective support and decisional control.
C.	Questions about overall satisfaction with the call.
D.	Questions that describes the call as well as the caller.

The TISS scale, developed and validated by Mattisson et al. [15], is a part of the TISQ, and was not developed for the present study. The TISS scale has demonstrated good psychometric properties, including high internal consistency (ordinal alpha 0.82–0.97) and satisfactory test-retest reliability (ICC 0.77–0.86). The TISS scale are divided across four different subscales based on the four components of the IMCHB: health information (eight questions), professional-technical competence of the telenurse (five questions), affective support (nine questions) and decisional control (three questions). See Mattisson et al. [15] for a full list of the 25 questions. For twenty of the questions, respondents select a response on a 4-point scale ranging from “Yes, completely” to “No, not at all”. The remaining five responses uses a five point-scale, from “Very satisfied” to “Very dissatisfied”. When the survey is compiled, the TISS as a whole and its four subscales are transformed into a ten-point scale, in which higher scores correspond to greater satisfaction with the interaction. As part of this process, it is necessary to first reverse the scores for the different questions, since the TISS scale gives low scores for positive response and high scores for negative response. The following formula is then applied: ((raw scale score − lowest possible score)/possible score range) × 10 [15].

In total, 286 questionnaires were distributed and 125 responses received, resulting in a total response rate of 43.7% (see Table 2 for the number of questionnaires sent and responses received from each participating region).

**Table 2:** Number of questionnaires sent and responses received from each participating region

Region/data collection period	Sent questionnaire/Received responses (response rate)
Region 1/2022	138/63 (45.7%)
Region 2/spring 2022– spring 2024	82/38 (46.3%)
Region 3/spring 2022– spring 2024	66/24 (36.4%)
Total	286/125 (43.7%)

Twelve responses were excluded, either because the parent’s child was over five years of age (*n* = 6) at the time of participation, or because age information was lacking (*n* = 6). There were thus 113 usable responses in total.

#### Qualitative data collection

In the questionnaire, 57 parents consented to be contacted again with information about a follow-up interview. These parents were contacted via SMS or email, depending on their preference. Twelve parents agreed to participate in the interview and provided written informed consent after receiving both written and oral information about the study, similar to the information they received when they filled in the questionnaire.

Semi-structured interviews were conducted by the first author, via video call. The interviews began with an open question: *“Tell me about the situation that made you contact the telephone nursing service”.* Thereafter, questions about various aspects of the interaction were posed according to an interview guide. The questions were based on the aim of the study (see additional file 2), along with follow-up questions that encouraged the parents to elaborate on their answers; for example: *“Can you tell me more about … ?”* and *“Elaborate on what you mean when you say …”*. Two pilot interviews were performed, leading to the elimination of one of the interview questions, *“How do you view your own knowledge when it comes to children and fever”.* This question could be negatively perceived by parents, and the answers it elicited did not contribute any information valuable to the aim of the study. Both pilot interviews were included in the data analysis since they otherwise served the aim of the study. Overall, the interviews provided diverse perspectives. In the later interviews, the answers were consistent with earlier ones, indicating the material adequately captured the phenomenon. With the parents’ consent, the interviews were audio-recorded and thereafter transcribed verbatim by the first author. The lengths of the interviews varied between 17-39 minutes, with at total time of 318 minutes (mean time 26.5 minutes).

### Data analysis

#### Quantitative data analysis

Descriptive statistics. The collected variables were analysed using the statistics software SPSS Version 30.0.0 (IBM Corp, 2024.). The findings are presented using absolute and relative frequencies (n, %). The level of satisfaction is presented using median (Md) and interquartile range (IQR).

#### Qualitative data analysis

The transcribed interviews were analysed using manifest content analysis, as described by Erlingsson and Brysiewicz [16]. With a focus on the purpose of the study, the transcribed interviews were read through repeatedly by the first author, in order to get a sense of the content as a whole. Thereafter the first and last authors selected two interviews, which they each analysed separately by extracting meaning units (i.e. words, sentences or pieces of text the content of which could be tied to the study’s aim). These units were condensed into condensed meaning units and then coded individually. The results from both authors were subsequently compared. Following this initial comparison, the first author applied the same procedure to the remaining interviews. Finally, the first and the last authors reviewed all codes to ensure they accurately reflected the material, and the codes were jointly organized into subcategories and categories. Throughout the different steps, all authors met continuously and reflected on the findings. The data analysis was not linear, but rather involved moving back and forth between the steps as part of a continuous process of reflection regarding whether the data analysis captured what the parents expressed in the interviews [16]. For examples of the qualitative data analysis process, see Table 3.

**Table 3:** Examples of the qualitative data analysis process

Meaning unit	Condensed meaning unit	code	Subcategory	Category
*“She took the time … to talk to us and listen”* – F6	Took the time to talk and listen	The telenurse allocates time	Being heard	Getting a caring conversation
*“Yes, well, the thing was that she, yeah, it felt like she took me seriously” – K11*	Feeling of being taken seriously	The telenurse takes the caller seriously	Being taken seriously	
*“Because she showed us empathy during the conversation – I mean, you could hear it in her voice” – J10*	You could hear empathy in her voice	An empathetic voice	Having a conversation with a sympathetic nurse	

#### Integrated data analysis

The integrated data analysis was carried out according to the steps described by Creswell and Plano Clark [13] for convergent design. Following analysis of both data sets, they were merged into a common result. First, similar concepts from both sets were identified and a joint presentation format was developed to facilitate an easy comparison. Thereafter, the common concepts were compared in order to determine whether they confirmed, expanded upon, or contradicted each other. Finally, this information was interpreted to provide insight into the research question.

### Ethical considerations

Ethical approval was obtained from the Swedish Ethical Review Authority (Ref. no.: 2020–03731 and 022–04587-02). In line with the principles of the Declaration of Helsinki [17], all participants obtained written information about voluntary participation and the opportunity to ask questions. Informed consent was given by the participants when completing the questionnaire and the parents who participated in an interview gave additional written consent. The collected data were kept confidential, in order to protect the participating parent’s privacy and integrity. When transcribing the interviews, they were pseudonymised and assigned an identification code for the data analysis process. Only the first author had access to the participants identities.

## Results

The results were divided into three parts, i.e. The quantitative results, the qualitative results and the integration of the two results, which are presented in an overall interpretation.

### Quantitative results

The respondents were primarily healthy 26– to 44–year-olds (93.8%) with a university or college degree (61%). A majority were parents to children aged 1–2 years (52.3%). Responses were most frequently provided by women (77.9%). All participants spoke Swedish during their TN interaction, though 9.7% stated that Swedish was not their first language. For detailed demographic data, see additional file1.

**Fig. 2:**
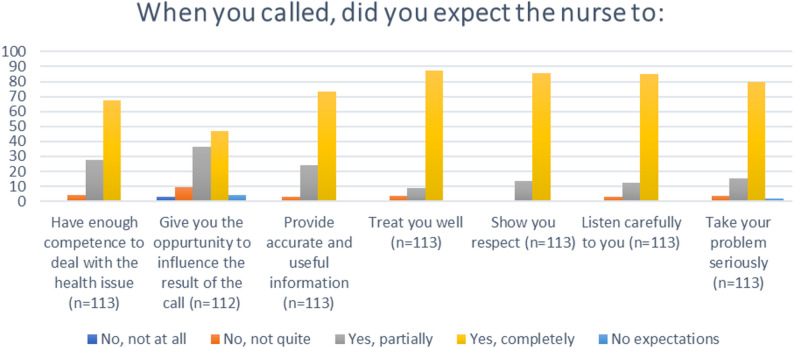
Parents’ expectations of the telenurse prior to the call

When asked about expectations prior to the call, almost all the parents expected to be completely or partially taken seriously, to be listened to, be respected, and to be treated well by their telenurse. They also expected the telenurse to provide accurate and useful information and to have sufficient competence to answer their questions. Less than half answered “yes, completely” when asked whether they expected to be given the opportunity to influence the result of the call, see Fig 2.

The majority of the calls occurred out of hours (67, 59.3%), and most (103, 91.5%) of the parents stated that they urgently/very urgently needed to get in touch with the telenurse. About two thirds (75, 66.4%) rated their level of concern as not at all concerned/somewhat concerned, and 53.9% perceived the waiting time to be slightly too long/far too long. The parents knew/had an idea of what they wanted (96, 86.7%), and the most common request was to receive self-care advice from the telenurse. The majority of parents (101, 89.4%) expected to fully/partially get what they wanted. The parents considered the length the conversation to be just right. As a result of their call, 64.6% of the parents got self-care advice from the telenurse, 92.1% were satisfied/very satisfied with the outcome and 98.2% stated that their expectations prior to the call where completely/partially met. A few stated that a discussion about lifestyle factors and/or preventive measures were included in the telenursing advice they received. A majority wanted to talk to the same telenurse again in future calls to the TN service, stated that they were satisfied or much satisfied with the call overall, and expressed satisfaction with previous calls to the TN service; see Table 4.

**Table 4:** Information about/results of the call and the parents’ wishes and expectations

(*n* = 113)	n (%)
Time of the call	
-Weekday, office hours (8–17:00)	46 (40.7)
-Saturday, Sunday or public holiday, daytime (8–17:00)	27 (23.9)
-Evening or night time (17–08:00)	40 (35.4)
Caller’s perceived level of urgency when calling	
-Not urgent at all	4 (3.5)
-Fairly urgent	6 (5.3)
-Urgent	50 (44.2)
-Very urgent	53 (46.9)
Caller’s perceived level of concern	
-Not at all concerned	6 (5.3)
-Somewhat concerned	69 (61.1)
-Concerned	24 (21.2)
-Very concerned	14 (12.4)
Perceived waiting time	
-Did not have to wait	3 (2.7)
-It was acceptable	49 (43.4)
-It was slightly too long	30 (26.5)
-Far too long	31 (27.4)
Most desired result of the call?	
-Self-care advice from the telenurse	88 (77.9)
-Appointment with GP on call	12 (10.6)
-Advice to go to the emergency department	6 (5.3)
-Advice to contact another caregiver	7 (6.2)
How strong was this desire when calling?	
-Knew exactly what they wanted	46 (40.7)
-Had an idea of what they wanted	51 (46.0)
-Unsure of what they wanted	15 (13.3)
Did you expect to get what you wanted?	
-Yes, completely	45 (39.8)
-Yes, partially	56 (49.6)
-No, not quite	6 (5.3)
-No, not at all	1 (0.9)
-Had no expectations	5 (4.4)
Perception of conversation length	
-The conversation was the right length	111 (98.2)
-The conversation was too short	1 (0.9)
-The conversation was too long	1 (0.9)
The result of the call	
-Self-care advice from the telenurse	73 (64.6)
-Appointment with GP on call	14 (12.4)
-Advice to go the emergency department	8 (7.1)
-Advice to contact another caregiver	18 (15.9)
Satisfaction with the result of the call	
-Very satisfied	76 (67.3)
-Satisfied	28 (24.8)
-Neither satisfied or dissatisfied	6 (5.3)
-Dissatisfied	1 (0.9)
-Very dissatisfied	2 (1.8)
Overall, did the call meet your expectations?	
-Yes, completely	87 (77.0)
-Yes, partially	24 (21.2)
-No, not quite	1 (0.9)
-No, not at all	1 (0.9)
-I had no expectations	0 (0)
Discussed lifestyle factors and/or preventive measures	
-Yes	17 (15.0)
-No	96 (85.0)
Would you like to speak with the same telenurse again?	
-Absolutely	100 (88.5)
-Maybe	10 (8.8)
-Preferably not	1 (0.9)
-Absolutely not	0 (0)
-Don’t know	2 (1.8)
Overall satisfaction with the call	
-Very satisfied	83 (73.5)
-Satisfied	26 (23.0)
-Neither satisfied or dissatisfied	3 (2.7)
-Dissatisfied	1 (0.9)
-Very dissatisfied	0 (0)
Overall satisfaction with previous calls to 1177	
-Very satisfied	39 (34.5)
-Satisfied	55 (48.7)
-Neither satisfied or dissatisfied	11 (9.7)
-Dissatisfied	8 (7.1)
-Very dissatisfied	0 (0)
Overall satisfaction with previous interactions with healthcare providers	
-Very satisfied	30 (26.5)
-Satisfied	56 (49.6)
-Neither satisfied or dissatisfied	16 (14.2)
-Dissatisfied	9 (8.0)
-Very dissatisfied	1 (0.9)
-Had no prior contact	1 (0.9)

**Fig. 3:**
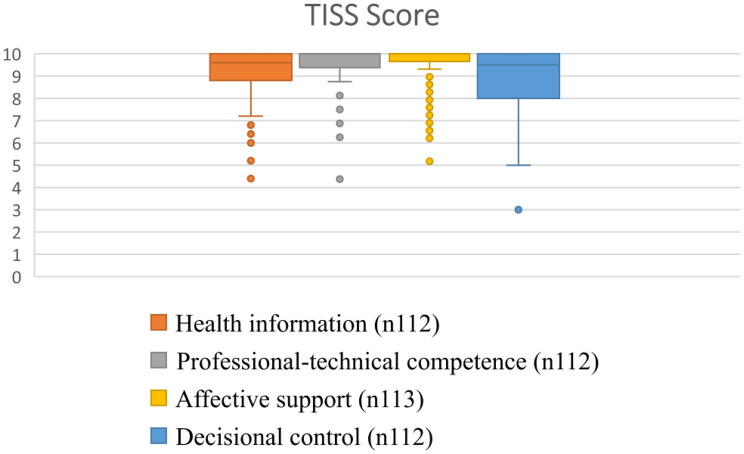
The parents rated satisfaction on TISS four subscales

The parents’ ratings indicated high satisfaction with their interaction with the telenurse. Affective support (Md 10, IQR 5.2–10) and professional-technical competence (Md 10, IQR 4.4–10) rated slightly higher than health information (Md 9.6, IQR 4.4–10) and decisional control (Md 9.5, IQR 3–10); see Fig. 3.

### Qualitative results

The twelve parents who participated in an interview, nine mothers and three fathers, were aged 26–40 (mean age 34). The children’s ages ranged from 1.5 months to 5 years at the time of the TN call.

**Fig. 4:**
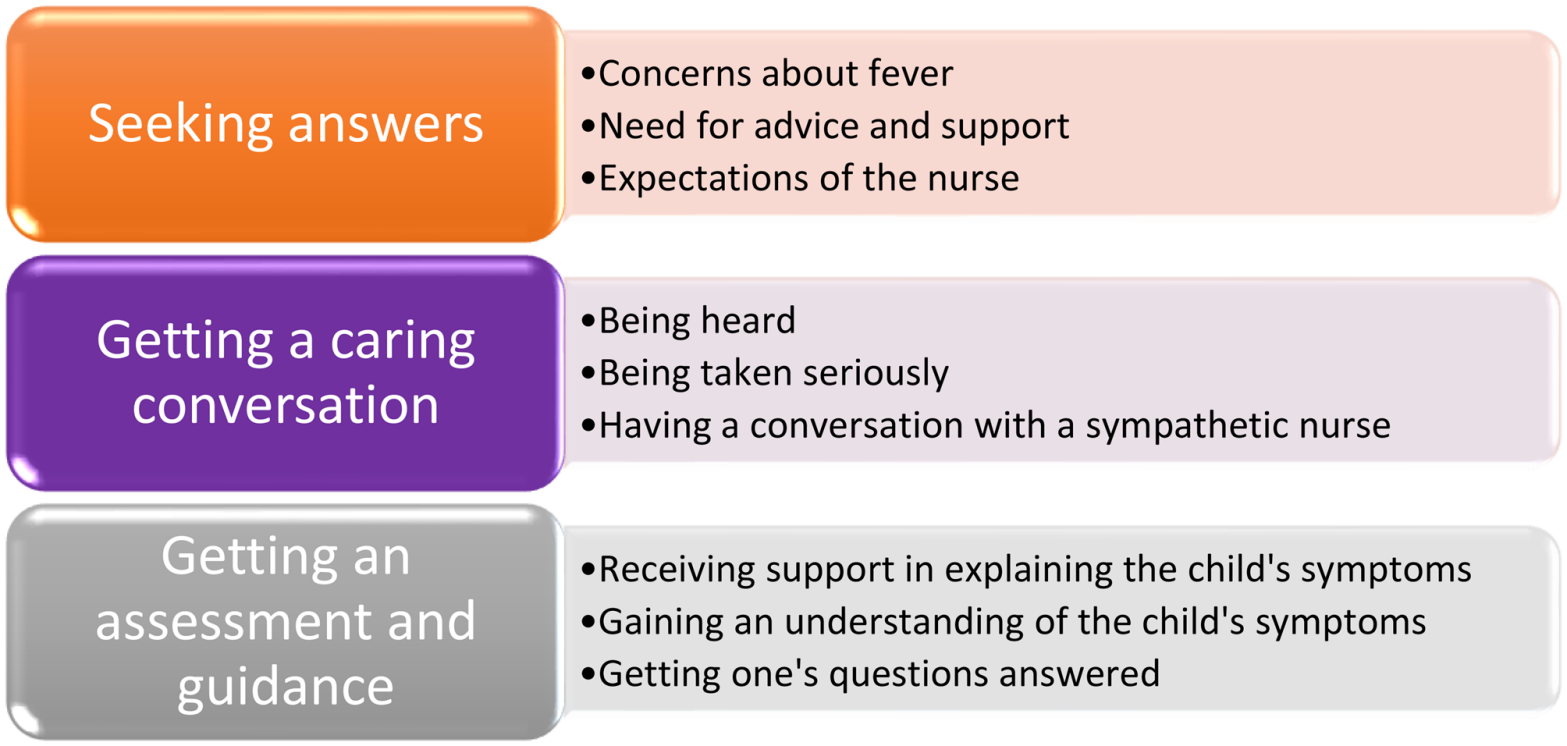
Overview of categories and subcategories that emerged in the qualitative analysis

In the qualitative analysis, three main categories emerged: seeking answers, getting a caring conversation, and getting an assessment and guidance. Each category contains three subcategories; see Fig. 4.

#### Seeking answers

When their child has a fever, the parents describe seeking answers about their child’s situation and symptoms.

##### Concern about fever

The parents describe different aspects of their child’s fever that motivated them to contact the TN service. For example, they did so because their child had a persistent fever, the fever presented differently than usual, their child lacked other symptoms, or their temperature increased rapidly and did not fall despite the fact that they administered antipyretics. The parents perceive fever as unpredictable and distressing. Even though they possess general knowledge about fever, they describe that specific symptoms tied to fever can cause concern. Their degree of concern varies from simple questions on how to monitor their child at home to imagining a worst-case scenario in which they will need to seek care at the paediatric emergency department.

*”Fever often comes before all other symptoms, so you’re not really sure what causes it or how it will develop. So, it’s quite … it could be quite scary, especially if it just spikes and suddenly they get completely lethargic”* – H8

#### Need for advice and support

The parents contact the TN service to get accurate and reliable answers. They want an assessment of their child’s symptoms and guidance on how to act, both in terms of self-care (such as dosage of antipyretics), as well as direction on if and when they should visit primary care or the paediatric emergency department. The parents also express a need to discuss their thoughts, in order to ensure they are making the right decisions – regardless of whether or not they wish to remain at home. Telenursing is considered an entry point to healthcare prior to turning to other parts of the healthcare system.

*”You don’t want her [the child] to have something where someone else who has a bit more experience will say, like, ‘Well, you should probably seek [medical care] for this. You want to have someone to ask, someone who’s experienced in this, unlike myself, so perhaps you can get a bit of advice and someone to bounce ideas off.” –* L12

Parents consider it important to receive prompt contact and clear answers to their questions. They express that long waiting times can exacerbate their existing concerns.

#### Expectations of the nurse

Parents expect to talk to a professional, knowledgeable and experienced telenurse who can adapt their advice to the situation at hand. They also expect to receive adequate and knowledge-based advice that remains consistent, regardless of whom the parent speaks to. Telenurses are also expected to be friendly and listen, as well as be able to handle the concerns that a child’s fever can cause.

*“I was hoping that I could, like, get … what’s it called? … a bit of reassurance. That they could calm my nerves or obsessive worrying.” –* A1

Not all parents have positive expectations; in the interviews, uncertainties emerged regarding the telenurses’ ability to help.

#### Getting a caring conversation

The parents describe different aspects of their interactions that reinforced the sense that their telenurse really cared.

#### Being heard

The telenurses are largely described as attentive and genuinely interested in hearing what the parents had to say. This is evident from the fact that the parents were allowed to speak without being interrupted or having to say things twice. Other signs that the telenurse was listening included their reiteration of what a parent had said, that they asked relevant follow-up questions, and that they provided answers based on what the parent had told them. It is regarded as important that the telenurse remain calm and professional, even if a parent shares serious concerns or worries that their child’s condition could be serious. By not acting stressed or trying to end the interaction too quickly, the telenurse shows that they have time for the parent who has called them. This gives parents a sense of comfort, makes them feel prioritised, and instils confidence that the telenurse is doing a thorough assessment.

*”She was very calm, and listened and asked questions and repeated what I had said and stuff like that, so I could tell that she really wanted to understand what I had said … it felt like we got to talk about everything, and that we’d be done when we were done, whether that took 10 minutes or 30 minutes – it didn’t feel like it mattered.”* – E5

#### Being taken seriously

Telenurses are seen as taking parents and their concerns seriously. This is appreciated, especially by parents who find that they themselves are more concerned than their child’s condition requires. The parents welcome hearing that it was good that they contacted the TN service. This prevents them from feeling as if they are taking up the telenurses time unnecessarily. They also appreciate it when the telenurse bases their assessment and advice on the parents’ concerns, rather than solely on the child’s condition. Even if the parents themselves might express that their child “just has a fever”, they make it clear that they would not feel reassured if a telenurse were to talk that way. If they do not feel they are taken seriously, parents’ concerns may worsen.

*Yes, she was still serious about the situation, like, even though there wasn’t really anything serious going on with him, other than a high fever and a cough. It was still as if she could feel me, could empathise that as a parent, of course you get concerned when your child gets a high fever and a cough* – *and she said several times that it was really good that I called.*– K11

#### Having a conversation with a sympathetic nurse

The telenurses are described as nice and friendly, often based on their voice and tone. Engaging in light conversation is reassuring. The telenurses are further described as understanding and empathetic. Showing interest, taking time to call a parent back, consulting the paediatric emergency department, and ensuring at the end of the interaction that the parent is satisfied – all these actions create a sense of helpfulness and make it seem that the telenurse genuinely has the best intentions. This, in turn, conveys a sense of calm and reassurance. The parents appreciate being praised for the way they handled their child’s fever, especially since they might fear doing anything wrong. A friendly approach can outweigh inadequate advice.

*”I knew that I had gotten the wrong advice, but I still felt like, ‘Oh, well* – *it will work itself out … It was still a good conversation. I thought she was nice, and I felt that I’d been treated with kindness and understood somehow, so that made me a bit more forgiving.”* – H8

#### Getting an assessment and guidance

The parents describe how the telenurses make an assessment, provide information, and answer questions.

#### Receiving support in explaining a child’s symptoms

The telenurses ask a lot of questions. This is viewed by the parents as a way to get a clear picture of the situation and the child’s condition. By asking questions, the telenurse is seen as helping the parents to adequately describe their child’s symptoms. This is considered important, because parents can feel uncertain about which information they should provide. Since there are numerous reasons for a child to have a fever, parents want their telenurse to adopt a broad perspective, even if the parents have zeroed in on a particular diagnosis. A mother with Swedish as a second language highlights how important it is that a telenurse asks concise and easily comprehensible questions, in order to avoid misunderstandings.

*“They [telenurses] need to ask, like, relevant questions, so that you doesn’t just ramble on and start talking about, like, what she ate the day before yesterday and whether that could be a reason [for the child having a fever]– B2*

#### Gaining an understanding of the child’s symptoms

Telenurses gave both general information on fever and symptom management, as well as guidance on how to act. For parents, it is important to be presented with a plan, because knowing what they should do to help their child can decrease anxiety. Information and guidance can provide reassurance, both in the moment and going forward, provided that it is adjusted to the demands and needs of the child and their parents. It is calming when a telenurse explains symptoms, such as the fact that it is common for a feverish child to have a rapid pulse and respiratory rate, or when the telenurse points out that a child’s symptoms have improved and talks about which symptoms can be serious. It is also reassuring when the telenurse refers to other parents whose child has similar symptoms, thus indicating, e.g. that a common virus is circulating.

*She [the telenurse] pointed out several times that kids can get them [high fevers], because he had a fever of nearly 41 degrees, and it was, like, that it’s not very worrying, but children can get that, and it’s not really strange for it to be that high, it’s not necessarily anything serious. And of course that was good to hear.*– K11

#### Getting one’s questions answered

The parents describe feeling reassured after their call to the TN service. They feel safer having talked to an expert and gotten their questions answered. When they do not receive answers, they feel they must turn elsewhere, but are uncertain of where. In some cases, the parents got other directions when, after talking to a telenurse, they contacted primary care or the paediatric emergency department. This is perceived as confusing and undesirable, even if the advice they received does not necessarily seem wrong, and raises concerns about the telenurses’ knowledge and competence, but parents also express an understanding that every healthcare provider needs to make their own assessment.

*Of course, in an ideal world, we would have gotten the same advice from both places, so to speak … but after all that, I mostly think that the telenurse’s advice was, well, that it was probably wise to contact primary care, so they [the telenurse] could maybe have skipped contacting primary care and requesting an appointment* – B2

### Overall interpretation of the integrated results

The integration of the findings shows that the interviews both confirmed and expanded upon the questionnaire responses, providing a more comprehensive understanding of parents’ experiences of the TN service. A joint presentation of these integrated results can be found in additional file 3. All and all, the findings show a high level of parental satisfaction with telenurses. Parents turn to them for answers, but their interaction with the telenurse plays an important role and sometimes seems to play an even greater role in determining a parent’s satisfaction with the call than the actual advice they receive. When a child’s fever sparks concern and a desire to seek answers, parents turn to telenurses with their various needs, expectations and wishes. The parents often know what they want and have high expectations of the telenurse. Although the waiting times can be perceived as too long, the lengths of the calls are generally described as appropriate, not because of the duration itself, but because the telenurses seem calm and do not end the conversation prematurely. In such faceless meetings, there is a risk that parents could wind up feeling like the telenurse did not care about them and their specific circumstances, or that questions might remain unanswered. More often than not, parents describe being cared for by someone with an empathetic response including a reassuring tone of voice. By listening and steering the conversation with a steady hand and asking the necessary questions, the telenurse grasps the parent’s perspective on the situation. This is essential to provide reassuring advice.

## Discussion

This study aims to explore and describe parents’ experiences of telephone nursing when their child has a fever. The discussion is based on the integrated results.

In keeping with other studies [18, 19], overall, the findings indicate a high level of satisfaction with various aspects of telephone nursing. The ability to get advice by phone is often seen as positive, accessible and convenient [20–23], especially when access to primary care is limited [20]. Visits to the paediatric emergency department can be associated with long waiting times [21], and this generally creates frustration [22, 24]. Unfortunately, our study shows that long waiting times also can pose a problem in telephone nursing. They were a reason for dissatisfaction and sometimes exacerbated feelings of anxiety, which is not uncommon [25]. Waiting times have previously been shown to lower satisfaction with the TN service [8], and sometimes prompt parents to seek care elsewhere [22]. On the other hand, parents were satisfied with the length of their calls to the service. They describe that the nurses did not seem stressed or try to end the conversation too quickly, which made them feel prioritised. Kvilén Eriksson et al. [21] found that when telenurses invest time in parent callers, it can make them feel important. This aligns with the findings of our study.

The parents contacted the TN service to get answers and reassurance related to different aspects of a child’s fever, such as a high temperature or a fever that did not respond to antipyretics. Fever is known to cause parental concerns regarding both the potential seriousness of the underlying illness and complications related to fever, such as febrile seizures [4, 24–26]. High temperatures are often misinterpreted to be an indicator of severe disease or increased risk of harm, and sometimes these concerns elicit unrealistic fears about, e.g. brain damage [4, 25–27]. However, such serious concerns did not emerge in the present study, possibly because many parents with major concerns for their child’s well-being will contact the paediatric emergency department directly, rather than contacting a TN service [22]. Instead, the findings in the present study align with other research. Namely, they indicate that prolonged fever or the development of additional symptoms that make the situation more complex (such as a lack of energy to play or reduced appetite and fluid intake) [27, 28] will prompt contact with a healthcare provider. These symptoms can cause parents to start second-guessing themselves and their ability to handle the situation [27]. As in the present study, parents need validation that they are caring for their child correctly [4, 21, 26] and to be reassured that their child is not seriously ill [4, 22, 24, 25]. The findings in our study show that many parents expect telenurses to fulfil those needs.

The high satisfaction expressed in the present results is somewhat surprising, since the majority of calls resulted in self-care advice, which is usually tied to a lower level of satisfaction than referral to a healthcare provider [6, 8]. However, the majority of the parents wanted advice and support from their telenurse, rather than being referred to other healthcare providers. This suggests that that the telenurse’s fulfilment of a parent’s wishes and expectations are important for their sense of satisfaction. However, it should be taken into account that a majority of the parents stated that they had minimal or no anxiety prior to the call, and this may contribute to their satisfaction with self-care advice. Martinsson and Gustafsson [12] suggest that if the initial desire is to seek emergency care, the advice from the telenurse seems to have little or no significance. Similarly, Ali et al. [29] found that parents who experience high levels of anxiety before going to a paediatric emergence department are more likely to continue to feel uncertainty after the visit and to be less satisfied with the received care – which can lead to repeated contact with the healthcare service, since parents do not feel comfortable taking care of their child at home. This indicates that when it comes to creating a trusting relationship, the higher the degree of anxiety, the greater the demands of the interaction will be.

The interviewed parents were generally satisfied with the information and advice they obtained from their telenurse, especially with regard to how their child’s symptoms should be handled and how they could think mowing forward. Several described that it felt valuable to receive a clear plan, while others stated that they had received conflicting advice when they were referred onward to other healthcare services. These known problems align with previous findings [4, 22, 24]. Although the telenurses utilized a decision support tool, this requires a certain degree of interpretation, particularly in more complex cases, which fever can represent. In other parts of the healthcare system, for example at the paediatric emergency department, nurses rely mainly on their own knowledge and local guidelines. This may be one reason why parents receive conflicting advice [11]. Given that none of the parents in the present study referred to decision support, it remains uncertain whether they are aware that such tool is being used, or if they attribute the advice solely to the telenurses individual competence.

Parents commonly seek healthcare to get guidance on how to manage fevers [22, 24, 28], and one recurring request is that the advice provided should be clear and consistent [4, 22, 25, 28]. Information about the expected course of an illness and so-called “red flags” that should prompt a new interaction – i.e. s*afety netting* – have previously been found to reduce parents’ concerns [10] and decrease the number of follow-up visits within emergency healthcare [22].

The parents expressed being very satisfied with the care they received during the call. The telenurses are described as friendly, responsive and attentive, and are seen to take parents’ concerns seriously. For parents, being taken seriously is perceived as essential, irrespective of the level of care or whether they interact with a nurse or a physician [4, 10, 23, 24, 28]. Unfortunately, healthcare professionals do not always convey this impression, which can lead parents to feel they sought healthcare unnecessarily [28]. It is important to remember that those who reach out to the TN service are often are in a vulnerable situation, need help managing the situation, and are grappling with feelings of uncertainty [21, 23]. The parents repeatedly articulated a need to be reassured, even when they recognised that their worries were overblown. Halldorsdottir [30] emphasises that caring is a part of the nurse’s compassionate competence, which entails genuinely caring for both patients and relatives by being warm, open understanding and non-judgemental – attributes that facilitate an emphatic and supportive approach. Even if a child’s condition is not considered serious by healthcare professionals, the parents’ concerns must be addressed respectfully [31]. This requires a mutual understanding, a bridge between parent and nurse [30]. As Martinsson and Gustafsson [12] conclude, the need for care can certainly exist, even in the absence of a need for medical care.

## Strengths and limitations

A distinct strength with mixed studies is the combination of quantitative and qualitative data, which allows for a comprehensive understanding of parents’ experiences and triangulates methods in such a way that the different results reinforce each other [13]. It is considered a strength that the timeframe for data collection stretched over several infection seasons, but a limitation that no data were collected during summer. Another limitation is the exclusion of non-Swedish-speaking parents, only about 10% of the parents in this study spoke Swedish as a second language. As a result, language and cultural perspectives on contacting a healthcare provider when a child has a fever are not considered. The fact that there is no way to verify that all parental who sought telenursing advice were invited to participate is another limitation, there is a risk of bias, as it may be that mostly satisfied parents were invited or opted to participate. Nevertheless, the results reveal positive as well as negative aspects of telephone nursing calls. The lengthy data collection period reflects difficulties to recruit participants due to the COVID-19 pandemic and its significant burden on both healthcare and society. Using a validated questionnaire enhances the reliability and trustworthiness of the quantitative data. Descriptions of the study context and participants were provided, to enable readers to assess transferability to similar settings. Every step of the data collection and analysis processes were documented, to ensure dependability by showing a transparent process. The various steps of the analysis were discussed in the research group, which strengthens their credibility. Continuous reflection on prior perceptions (with the aim of minimising subjective bias) ensures confirmability, and the findings of each subcategory are echoed in accompanying quotes from participants.

### Conclusions and implications

The aim of this study was to explore and describe parents’ experiences of telephone nursing when their child has a fever. The findings show that TN encompasses both advice and the telenurse-parent interaction surrounding the provision of said advice. Both aspects are important for creating satisfied and reassured parents; the advice must be clear, consistent and preferably include safety netting. At the same time, telenurses must listen, validate and take parents’ concerns seriously.

When the telenurse brings together both their expertise and their ability of to care, TN can serve as a convenient alternative to visiting a healthcare facility, thereby limiting the strain on other parts of the healthcare system. One area for improvement is the discrepancies between the advice parents receive in various healthcare settings. Such variation creates confusion, and there is a need to review causes and potential solutions regarding this dilemma. Future research could focus on language and cultural perspectives on contacting a healthcare provider when a child has a fever.

## Data Availability

Deidentified data may be available on reasonable request via the corresponding author.

## Abbreviations

SHD: Swedish healthcare direct
TN: Telephone nursing
IMCHB: Interaction model of client health behaviour
TISQ: Telenursing interaction and satisfaction questionnaire
TISS: Telenursing Interaction and Satisfaction Scale
